# Predictors of outcome for treatment of enterovaginal fistula: Therapeutical strategies for treatment

**DOI:** 10.1007/s00384-023-04453-2

**Published:** 2023-07-07

**Authors:** Moritz Drefs, Sebastian Schömer Cuenca, Ulrich Wirth, Florian Kühn, Maria Burian, Jens Werner, Petra Zimmermann

**Affiliations:** 1 Department of General, Visceral and Transplant Surgery, University Hospital of Munich, Munich 81377, Germany; 2 Klinikum Freising, Freising, Germany

**Keywords:** Enterovaginal fistulas, Rectovaginal fistulas, Therapeutic outcome for enterovaginal fistulas

## Abstract

**Background:**

Enterovaginal fistulas represent a serious complication of various diseases and therapeutic procedures, often associated with complicated clinical courses and massive impairment of quality of life. As underlying conditions and procedures are multifarious, therapeutic approaches are challenging and have to be tailored individually. As the therapeutic management is complex and individualized, multiple surgical interventions might be necessary.

**Methods:**

The aim of this study was to identify possible predictors for outcome in the treatment enterovaginal fistula patients. The study was realized as a retrospective analysis. Ninety-two patients treated with enterovaginal fistulas between 2004 and 2016 were analyzed. Patient characteristics, therapeutic data, and endoscopic findings were stratified according to etiology, closure rate and time, as well as recurrence of fistula. Main outcome measure was the overall rate of fistula closure.

**Results:**

Overall therapeutic success rate was 67.4%. Postoperatively derived fistulas were most frequent (40.2%), mainly after rectal surgery (59.5%). Postoperative and non-IBD-inflammation associated fistulas had better outcome than IBD-, radiotherapy-, and tumor-related fistulas (*p* = 0.001). Successful fistula closure was observed more frequently after radical surgical interventions, best results observed after transabdominal surgery (*p* < 0.001). Fistula recurrence was also less frequently observed after radical surgical therapies (*p* = 0.029). A temporary stoma was associated with higher incidence of fistula closure (*p* = 0.013) and lower incidence of fistula recurrence (*p* = 0.042) in the postoperative subgroup, as well as shortened therapy period in all groups (*p* = 0.031).

**Conclusion:**

Enterovaginal fistulas are a result of various etiologies, and treatment should be adjusted accordingly. A very sustainable, rapid, and persistent therapeutic success can be expected after radical surgical approaches with temporary diverting stoma. This is especially true for postoperatively derived fistulas.

## Introduction

Enterovaginal fistulas are rare afflictions possibly leading to severe clinical symptoms with critical impairment of quality of life for affected women. A variety of predisposing conditions and surgical procedures have been described to potentially cause enterovaginal (EV) fistulas [1–4, 9–11]. The postpartum development of entero- or rectovaginal fistulas is most common in up to 88% of all EV fistula events [1, 2]. Crohn’s disease with perineal disease manifestation causes only approximately 0.2–2.1% of all enterovaginal fistulas [2, 9]. Yet, the rate of Crohn’s disease-related EV fistulas might rise up to 10% in case of previous rectal resections. Likewise, entero- or rectovaginal fistulas are a relevant phenomenon after rectal surgery for malignant and benign diseases as well as after pouch procedures in patients with ulcerative colitis and familial adenomatous polyposis [5, 7, 8]. Concurrently with the introduction of stapler hemorrhoidectomy and transanal stapled rectal resection for obstructed defecation, rectovaginal fistulas have been associated with these procedures [2–4, 6]. Likewise, surgical interventions using stapler devices for functional pelvic floor disorders have been reported to result in rectovaginal fistulas [2–4, 6]. Other than obstetric fistulas, gynecologic malignancies or radiation therapy for gynecologic tumors may lead to fistula development [10, 11].

As etiologic conditions and procedures vary wildly, therapeutic approaches are challenging and have to be individually tailored [14]. As the therapeutic management is complex and individualized, multiple surgical interventions might be necessary. Besides well-described local surgical approaches including mucosa flap, surgical repair might require a diverting stoma, rectal resection, or even complex reconstruction with muscle transposition [2, 12–18]. Nowadays, endoscopic advances such as OTS-clips (over the scope clips) offer new therapeutic prospects; yet, their clinical success and role has to be further evaluated [19].

While several studies have already focused on clinical features of selected EV fistula patients, data regarding the genesis and treatment proportions in EV fistula patients in real life settings is still scarce. Moreover, general conditions of the patients affected (e.g., comorbidities) should be taken into account when choosing treatment algorithm.

Therefore, the aim of the present study is to analyze long-term outcomes after various treatment modalities of EV fistulas of diverse origin and thus to identify possible predictors for treatment outcome.

## Methods

### Study design

The study was designed as a monocentric, observational cohort-study at a single academic reference center for Surgical Endoscopy. The study was approved by the Institutional Review Board of the LMU University of Munich (protocol number EK-LMU 19–062).

### Study population

**Fig. 1:**
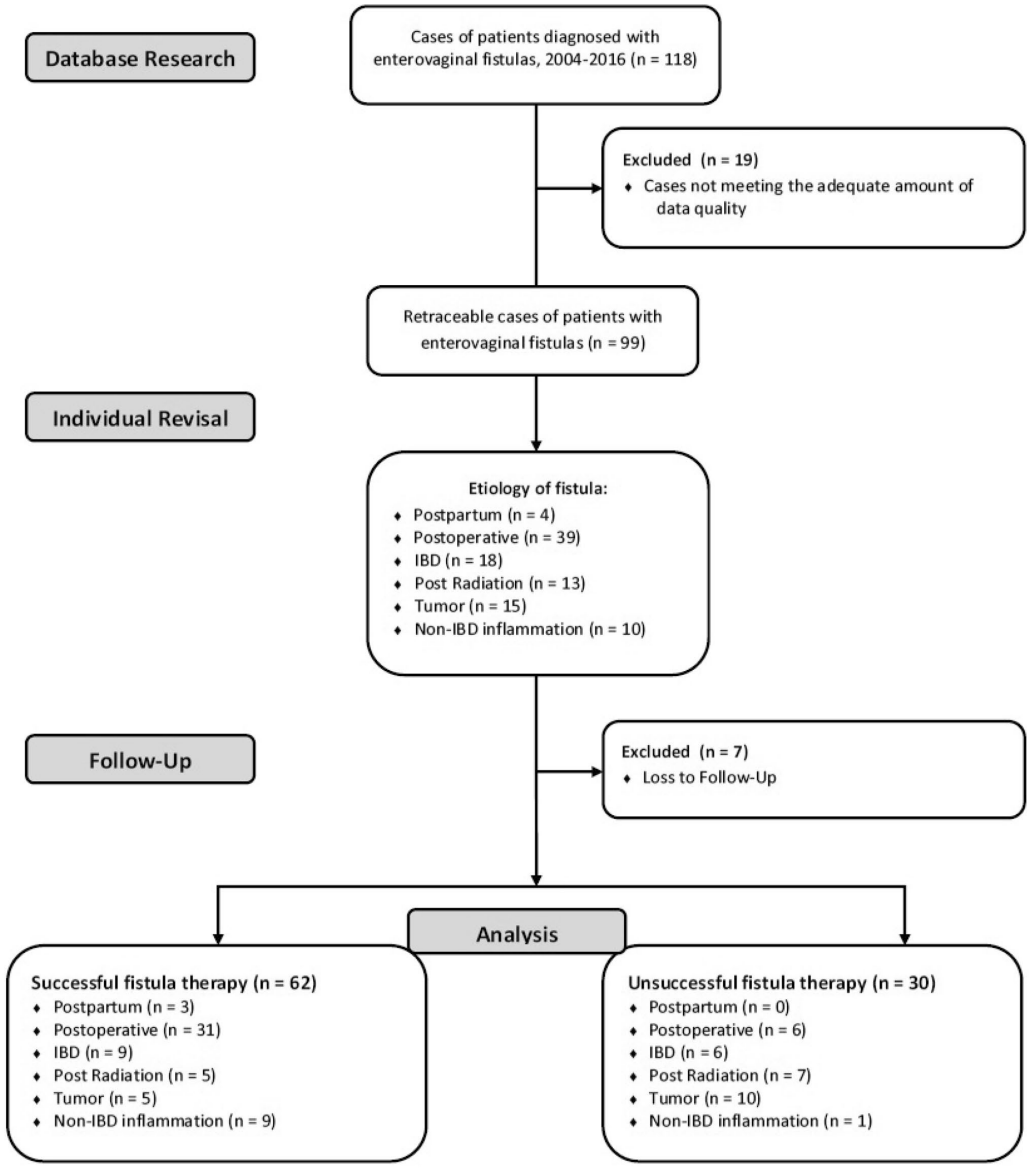
Flow diagram regarding the inclusion and exclusion criteria from database research to the final analysis of patient cohort with enterovaginal fistulas, treated at our institution. IBD, inflammatory bowel disease

One hundred eighteen patients who presented with entero-, colo-, recto-, or anovaginal fistula at the Department of General, Visceral and Transplantation Surgery of the Ludwig-Maximilians University Munich, between 01/2004 and 12/2016 were included into primary analysis (Fig. 1). These patients were stratified according to their respective fistula’s cause. Finally, patients were categorized into the successfully treated fistula cohort (SFT) if closure of the fistula was achieved during the observation period. If persistence of the fistula was detected during the observation period, respective patients were labeled as unsuccessful therapy cohort (UFT).

### Data sources

Demographic data, information on clinical history, clinical examinations, and therapeutic information were extracted from the clinical documentation system, clinical charts, endoscopic reports, and anaesthesiology reports.

### Outcomes

Overall rate of fistula closure was defined as primary endpoint. In addition, the following secondary endpoints are analyzed: rate of fistula closure depending on etiology, on therapeutic approach, and on existence of a diverting stoma; fistula recurrence after initial therapy success depending on etiology and treatment; and potential further influential factors on therapy success such as comorbidities.

Successful fistula closure was defined as missing clinical signs of enterovaginal fistula, meaning no secretion, no pus, and no newly occurred abscess. Unsuccessful treatment was defined as clinical evidence of fistula, e.g., persistent secretion, detectable fistula course, and newly occurred abscess.

### Patient- and treatment-specific variables

The following parameters were assessed at time of first fistula treatment: age, Charlson Comorbidity Index (CCI), serum albumin levels, preexisting anorectal diseases and previous surgeries, cause of EV fistula, antibiotic treatment, treatment modalities, number of therapeutic approaches applied per patient (therapy load), number of operations performed in curative intent (operative load), fistula recurrence after initial therapy success, and creation of a diverting stoma.

The Charlson Comorbidity Index (CCI) is an index to predict a mortality risk over time for comorbid conditions [20].

### Statistical analysis

This study was carried out as an explorative study. Descriptive statistical analysis was performed, and relations between fistula etiology, therapeutic success, and further underlying conditions were reviewed. In further steps inter-group differences were quantified by performing comparative analysis calculating Student’s *t*-test and Fisher’s exact test for single-group differences, as well as chi-square test for multiple group differences. Odds ratios have been calculated applying the Babtista-Pike method. For statistical analysis and graphical presentation, SPSS statistical software package (version 25, IBM, Chicago, Ill) and GraphPad Prism (version 8.4.2, GraphPad Software, San Diego, CA) were used. *p* value (two-tailed) of < 0.05 was regarded as statistically significant.

## Results

### Study population

Primary database query revealed a total of 118 patients who presented with enterovaginal fistula to our Surgical Endoscopy Unit between 2004 and 2016. 19 patients had to be excluded due to insufficient primary data, whereas 7 patients were excluded due to loss of follow-up. Hence, 92 patients (78%) were included into the final analysis. The algorithm of patient case selection for the analysis, as well as detailed number of patients stratified by fistula etiology is shown in Fig. 1.

Nineteen patients presented with an anovaginal fistula, 20 with a rectovaginal, and 12 with a sigmoidovaginal fistula. Two patients suffered from enterovaginal fistula, and for 39, a specific localization of the fistula origin within the colon and rectum was not documented. Median follow-up was 18.9 months.

The majority of patients was affected by postoperative fistulas (40.2%), and only 3.3% presented with postpartum fistulas. 59.5% of postoperative fistulas occurred after rectal resection, 27.0% after gynecological surgeries, and 13.5% after combined interventions. 44.6% of the patients did not have any history of pre-existing anorectal diseases. Of all postoperative fistulas, 12 (32.4%) occurred directly at the anastomotic site.

Overall closure rate was 67.4%, whereas fistula therapy was unsuccessful in 32.6%.

Details on primary therapeutic approaches as well as on fistula etiology and main demographic data are given in Tables 1 and 2 and Fig. 1.

**Table 1:** Overview on primary therapeutic approaches; resection might also have involved tumor resection at the same time. Information is given on the type of enteral resection

Therapeutic approach	*N* (%)
**Abdominal approach**	
Diverting stoma alone	13
Colonic resection with rectal stump and diverting stoma, secondary reconstruction	13
Rectal extirpation	4
Resection of anastomosis, new anastomosis ± diverting stoma	3
Low rectal resection with primary anastomosis ± diverting stoma	7
Sigma resection with primary anastomosis	4
Small bowel resection	2
Transabdominal suture of rectal and vaginal wall ± diverting stoma	4
**Perineal approaches**	
Conservative, seton-drainage	14
OTS-clip	4
Sphincter reconstruction, mucosa flap ± diverting stoma	14
Fistula excision	1
EndoVac	3
**Not documented**	6

**Table 2:** Description of characteristics and key variables of patient cohorts with enterovaginal fistula stratified for therapy success of fistula closure

Patient characteristics and control variables	All, *N* (%)/mean [95% CI]	Therapy success of fistula closure		*p* value (therapy success vs. failure)
		*–*SFT successful fistula treatment *N* (%)/mean [95% CI]	*–*UFT unsuccessful fistula treatment *N* (%)/mean [95% CI]	
﻿Age (y)	55.3 [52.1–58.4]	55.0 [50.8–59.2]	55.8 [51.4–60.2]	0.8143
**Etiology of fistula**				**0.0011**
Postpartal	3 (3.3%)	3 (4.8%)	0 (0%)	
Postoperative	37 (40.2%)	31 (50.0%)	6 (20.0%)	
IBD	15 (16.3%)	9 (14.5%)	6 (20.0%)	
Post radiation	12 (13.0%)	5 (8.1%)	7 (23.3%)	
Tumor	15 (16.3%)	5 (8.1%)	10 (33.3%)	
Non-IBD inflammatory	10 (10.9%)	9 (14.5%)	1 (3.3%)	
Total	92 (100%)	62 (67.4%)	30 (32.6%)	
**Etiology of postoperative fistula**				0.2625
Rectal	22 (59.5%)	17 (54.8%)	5 (83.3%)	
Gynecological	10 (27.0%)	10 (32.3%)	0 (0%)	
Combined	5 (13.5%)	4 (12.9%)	1 (16.7%)	
Total	37 (100%)	31 (83.8%)	6 (16.2%)	
**Preexisting anorectal diseases**				0.2225
IBD	15 (16.3%)	8 (12.9%)	7 (2.3%)	
Malign neoplasia	25 (27.2%)	17 (27.4%)	8 (2.7%)	
Benign neoplasia	1 (1.1%)	1 (1.6%)	0 (0%)	
Abscess	4 (4.3%)	3 (4.9%)	1 (3.3%)	
Diverticular disease	4 (4.3%)	4 (6.5%)	0 (0%)	
Colitis (non-UC)	2 (2.2%)	0 (0%)	2 (6.7%)	
None	41 (44.6%)	29 (46.8%)	12 (40.0%)	
**CCI-score**				
	3.2 [2.5–3.8]	2.8 [2.0–3.6]	4.0 [2.8–5.2]	0.0752
**Serum albumin levels at time point of primary fistula treatment**				
	3.8 [3.6–4.1]	3.8 [3.5–4.2]	3.8 [3.6–4.1]	0.9687
**Total**	92 (100%)	62 (67.4%)	30 (32.6%)	

*CCI* Charlson Comorbidity Index, *CI* confidence interval, *IBD* inflammatory bowel disease, *UC* ulcerative colitis, *y* years

### Therapy success and risk for recurrence depending on fistula etiology

**Fig. 2:**
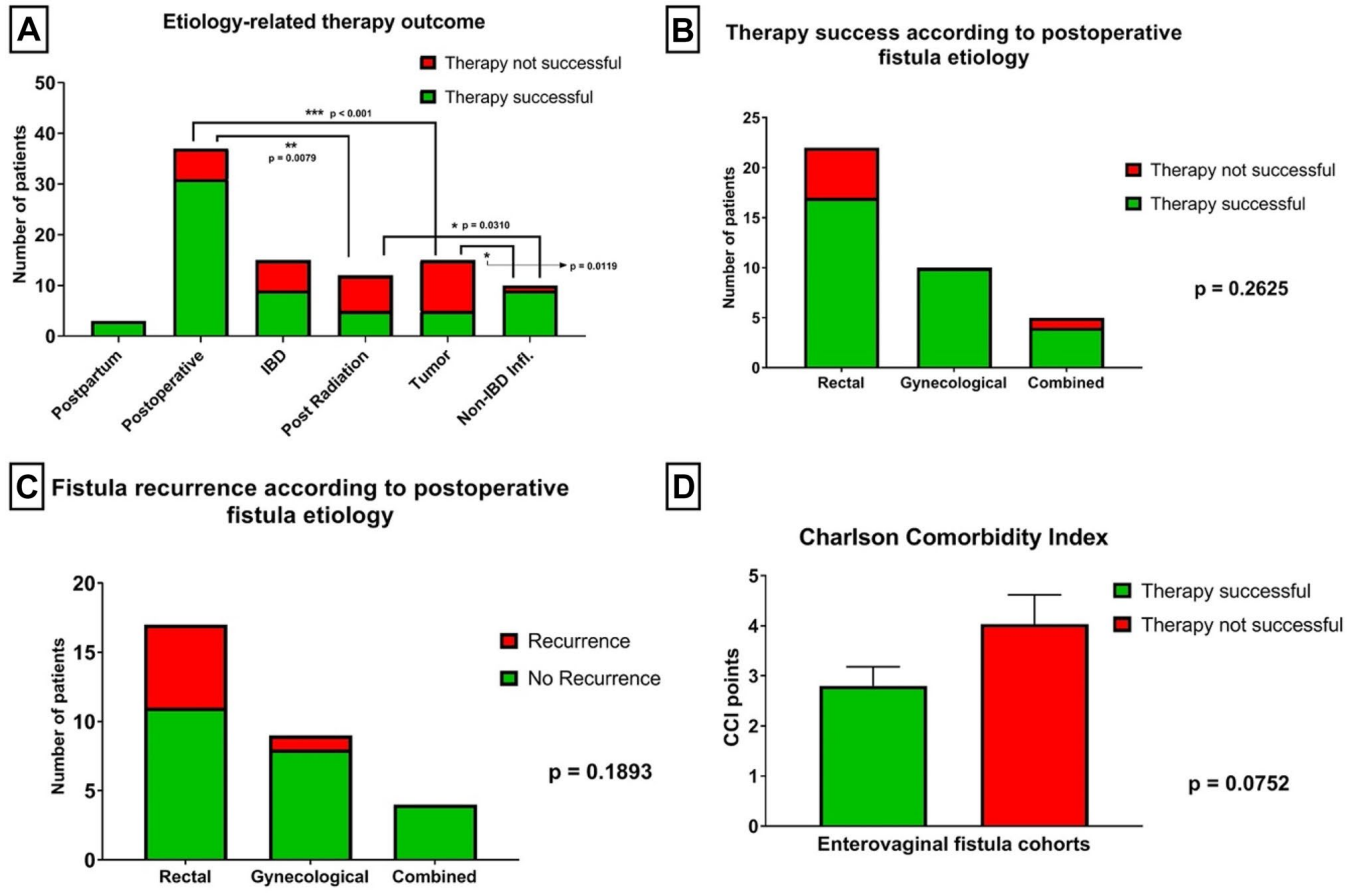
Outcome of fistula-specific therapy, stratified for etiology of fistulas and patient comorbidities. **A** Postoperatively derived enterovaginal fistulas showed a significantly more favorable therapy success rate than fistulas associated with tumor (*p* < 0.001) and radiation (*p* = 0.079). Non-IBD inflammatory fistulas were treated more successfully than fistulas associated with tumor (*p* = 0.0119) and radiation (*p* = 0.0310). No statistically significant differences in therapy outcome were monitored between fistulas after rectal, gynecological, or combined surgery, yet with a favorable tendency for gynecological pre-operation (**B**, **C**). **D** Patients with a CCI score were associated with a more unfavorable outcome after fistula treatment, yet not significantly (**D**; *p* = 0.0752). IBD, inflammatory bowel disease

Concerning fistula etiology, postoperative and non-IBD inflammatory fistula (e.g., diverticulitis) had the best therapeutic outcome, which was significantly better as compared to fistula of other etiologies (Fig. 2A). Postpartum fistulas showed a 100% therapeutic success. Tumor- and radiotherapy-related fistulas demonstrated the least favorable outcome with a high proportion of unsuccessful therapies. Likewise, IBD-related fistulas were related to impaired therapeutic outcome.

No difference could be detected between etiology-specific subgroups regarding risk for fistula recurrence. Concerning postoperative fistulas, there was a tendency towards better results in case of primary gynecological surgeries compared to primary rectal resections leading to enterovaginal fistulas, though the groups were small, and the comparison did not reach statistical significance (*p* = 0.1893; Fig. 2C).

Details on therapy outcome with regard to fistula etiology are given in Table 2 and Fig. 2.

### Therapy success depending on co-morbidities

A higher CCI-score was associated with a clear tendency towards poorer therapeutic outcome (*p* = 0.0752; Fig. 2D). However, no difference in serum albumin-levels was detected between the SFT and UFT patient cohorts. Pre-existing anorectal disorders did not show any influence on therapeutic outcomes (Fig. 2B). Details are shown in Table 2 and Fig. 2.

### Therapy success depending on type of therapeutic approach

**Fig. 3:**
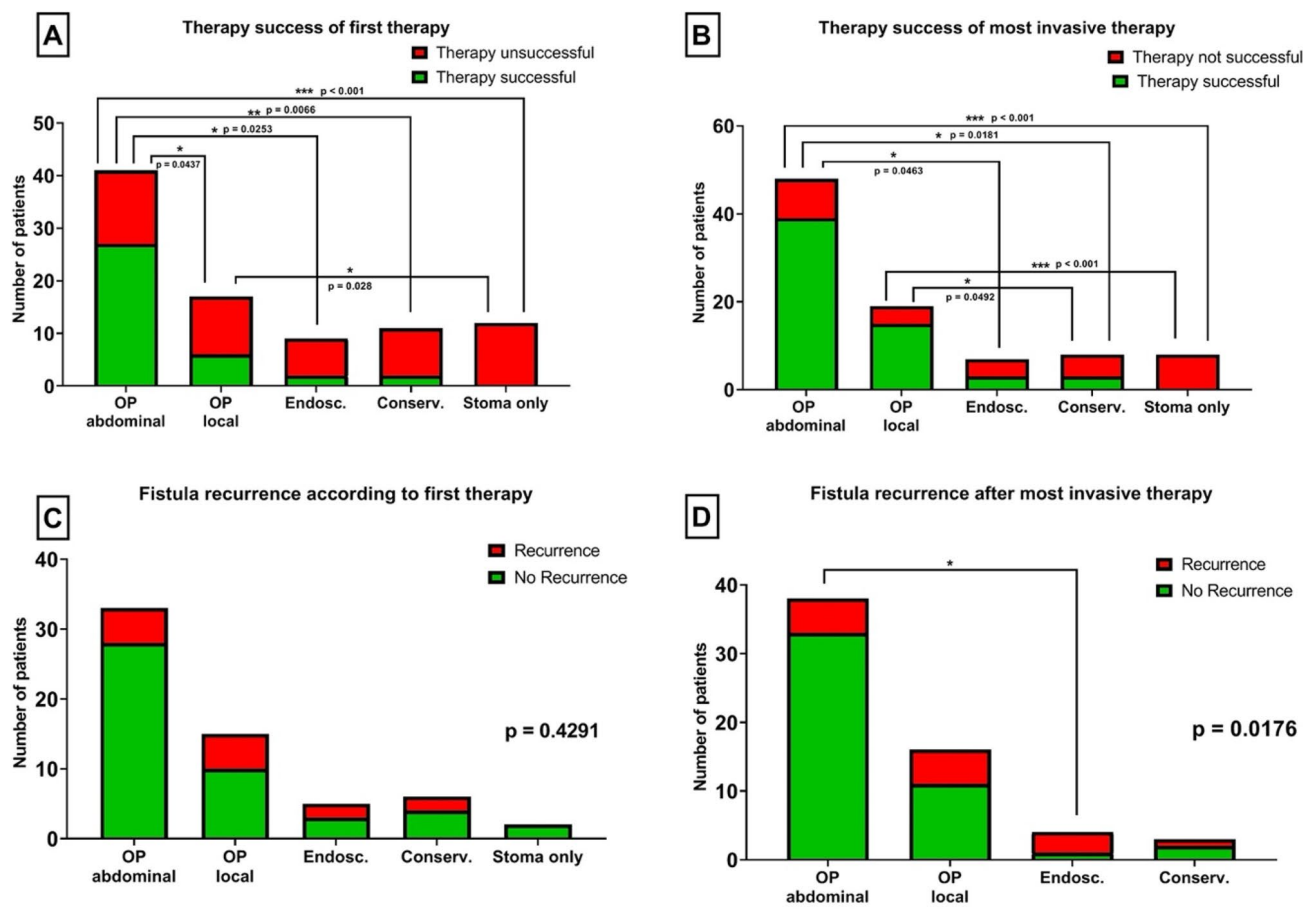
Outcome of fistula-specific therapy, stratified for applied treatment modalities. A transabdominal surgical approach for fistula treatment was associated with the highest primary success rate and therefore significantly better than any other applied therapy modality, when applied as first (**A**) or most invasive treatment (**B**) within the therapeutic sequence. Local surgical treatments still resulted in significantly better therapy outcome than the sole application of an ostomy as first (**A**) and most invasive treatment (**B**) and significantly better success rates than conservative treatment, when applied as most invasive treatment. **C** No statistically significant difference was monitored between the different applied first therapeutic options regarding fistula recurrence. **D** Significantly lower rates of fistula recurrence were observed after application of transabdominal surgery as the most invasive treatment within the therapeutic sequence. Conserv., conservative treatment; Endosc., endoscopic treatment; OP, surgery. *p* values as seen in figure

Regarding the first therapeutic approach, patients undergoing abdominal operation for fistula closure showed significantly better results with a closure rate of 69.2% as compared to local operative therapy, endoscopic therapy, conservative approach, or stoma alone (Table 3, Fig. 3A).

**Table 3:** Description of applied treatment modalities for enterovaginal fistulas, stratified for therapy success of fistula closure

Modality of fistula treatment	All, *N* (%)/mean [95% CI]	Therapy success of fistula closure		*p* value (therapy success vs. failure)
		SFT successful fistula treatment *N* (%)/mean [95% CI]	UFT unsuccessful fistula treatment *N* (%)/mean [95% CI]	
**Peritherapeutic antibiotic treatment**				> 0.9999
IV-AB treatment	44 (47.8%)	31 (50.0%)	13 (43.3%)	
No AB treatment	9 (9.8%)	7 (11.3%)	2 (6.7%)	
Unknown	39 (42.4%)	24 (38.7%)	15 (50.0%)	
**Primary treatment**				**0.0001**
OP–transabdominal	41 (44.6%)	27 (69.2%)	14 (26.4%)	
OP–local	17 (18.5%)	6 (15.4%)	11 (20.8%)	
Endoscopic	9 (9.8%)	2 (5.1%)	7 (13.2%)	
Locally conservative	11 (12.0%)	2 (5.1%)	9 (17.0%)	
Stoma only	12 (13.0%)	0 (0%)	12 (22.6%)	
Unknown	2 (2.2%)	2 (5.1%)	0 (0%)	
Total	92 (100%)	39 (44.6%)	53 (57.6%)	
**Most invasive treatment**				** < 0.0001**
OP–transabdominal	48 (52.2%)	39 (62.9%)	9 (30.0%)	
OP–local	19 (20.7%)	15 (24.2%)	4 (13.3%)	
Endoscopic	7 (7.6%)	3 (4.8%)	4 (13.3%)	
Locally conservative	8 (8.7%)	3 (4.8%)	5 (16.7%)	
Stoma only	8 (8.7%)	0 (0%)	8 (26.7%)	
Unknown	2 (2.2%)	2 (3.2%)	0 (0%)	
**Therapy load (number of therapy options applied)**				
	1.6 [1.4–1.8]	1.6 [1.4–1.8]	1.7 [1.4–2.1]	0.5237
**Operative load (number of operations performed)**				
	1.4 [1.2–1.5]	1.3 [1.2–1.5]	1.5 [1.0–1.9]	0.5237
**Total**	92 (100%)	62 (67.4%)	30 (32.6%)	

*AB* antibiotic, *CI* confidence interval, *IV* intravenous, *OP* operation

In case more than one treatment effort was needed due to fistula recurrence or persistence, a transabdominal approach as the most invasive treatment option still showed the best results with a closure rate of 62.9%. A local surgical approach also led to favorable closure rates (24.2%) (Fig. 3B). Moreover, a local surgical approach showed better closure rates than an endoscopic, conservative, or stoma alone treatment regimens.

Endoscopic interventions showed poor closure rates with only 5.1% success rate in case of primary approach and 4.8% in the overall analysis for all therapeutic options.

A diverting stoma alone was never sufficient for fistula closure. Yet, this approach was used for symptom control in case of tumor related fistula and a palliative setting.

There was no difference in fistula recurrence rates depending on the treatment chosen as the first curative treatment (Fig. 3C). Nevertheless, recurrence was monitored significantly less frequently in case of a more radical therapeutic approach at any time during the therapy sequence (Fig. 3D). Details on fistula closure rates and recurrence rates with regard to the therapeutic regimen are shown in Tables 3 and 4 and Fig. 3. Odds ratios for different therapeutic approaches are given in Table 5.

**Table 4:** Description of etiology and applied treatment modalities for enterovaginal fistulas, stratified for fistula recurrence

Etiology of fistula and respective treatment modality	All, *N* (%)/mean [95% CI]	Recurrence of fistula		*p* value (recurrence vs. no recurrence)
		Recurrence *N* (%)/mean [95% CI]	No recurrence *N* (%)/mean [95% CI]	
**Etiology of fistula**				0.9347
Postpartal	3 (4.8%)	0 (0%)	3 (6.3%)	
Postoperative	30 (48.4%)	7 (70%)	23 (47.9%)	
IBD	9 (14.5%)	2 (14.3%)	7 (14.6%)	
Post radiation	6 (9.7%)	2 (14.3%)	4 (8.3%)	
Tumor	5 (8.1%)	1 (7.1%)	4 (8.3%)	
Non-IBD inflammatory	9 (14.5%)	2 (14.3%)	7 (14.6%)	
**Etiology of postoperative fistula**				0.1893
Rectal	17 (56.7%)	6 (85.7%)	11 (47.8%)	
Gynecological	9 (30.0%)	1 (14.3%)	8 (34.8%)	
Combined	4 (13.3%)	0 (0%)	4 (17.4%)	
Total	30 (100%)	7 (23.3%)	23 (76.7%)	
**Primary treatment**				0.4291
OP–transabdominal	33 (53.2%)	5 (35.7%)	28 (58.3%)	
OP–local	15 (24.2%)	5 (35.7%)	10 (20.8%)	
Endoscopic	5 (8.1%)	2 (14.3%)	3 (6.3%)	
Locally conservative	6 (9.7%)	2 (14.3%)	4 (8.3%)	
Stoma only	2 (3.2%)	0 (0%)	2 (4.2%)	
Unknown	1 (1.6%)	0 (0%)	1 (2.1%)	
**Most invasive treatment**				**0.0294**
OP–transabdominal	38 (61.3%)	5 (35.7%)	33 (68.8%)	
OP–local	16 (25.8%)	5 (35.7%)	11 (22.9%)	
Endoscopic	4 (6.5%)	3 (21.4%)	1 (2.1%)	
Locally conservative	3 (4.8%)	1 (7.1%)	2 (4.2%)	
Unknown	1 (1.6%)	0 (0%)	1 (2.1%)	
**Total**	62 (100%)	14 (22.6%)	48 (77.4%)	

*CI* confidence interval, *IV* intravenous, *OP* operation

**Table 5:** Comparative analysis of different treatment regimens, stratified for both, primary closure and recurrence of enterovaginal fistulas

Comparison of therapeutic success influencing variables	Therapy success of fistula closure		Absence of fistula recurrence	
	Odds ratio [95% CI]	*p* value	Odds ratio [95% CI]	*p* value
**Primary treatment**				
Transabdominal vs. local	3.5 [1.1–11.2]	**0.0437**	2.9 [0.7–12.3]	0.2468
Transabdominal vs. endoscopic	6.8 [1.4–34.1]	**0.0253**	3.7 [0.5–21.4]	0.2227
Transabdominal vs. conservative	8.7 [1.7–42.5]	**0.0066**	2.8 [0.4–18.1]	0.2902
Local vs. endoscopic	1.9 [0.3–11.1]	0.6673	1.3 [0.2–8.3]	> 0.9999
Local vs. conservative	2.5 [0.4–13.8]	0.4188	1.0 [0.2–7.4]	> 0.9999
Conservative vs. endoscopic	1.3 [0.2–9.7]	> 0.9999	1.3 [0.1–12.3]	
**Most invasive treatment**				
Transabdominal vs. local	1.2 [0.4–4.0]	> 0.9999	3.9 [0.9–14.0]	0.1056
Transabdominal vs. endoscopic	5.8 [1.3–25.1]	**0.0463**	19.8 [2.3–261.3]	**0.0176**
Transabdominal vs. conservative	7.2 [1.4–29.8]	**0.0181**	3.3 [0.2–31.3]	0.3860
Local vs. endoscopic	5.0 [0.9–25.0]	0.1490	6.6 [0.7–91.9]	0.2553
Local vs. conservative	6.3 [1.0–29.7]	**0.0492**	1.1 [0.06–11.21]	> 0.9999
Conservative vs. endoscopic	1.3 [0.2–7.8]	> 0.9999	6.0 [0.3–111.1]	0.4857
**Supportive ostomy**				
Stoma vs. no stoma	1.7 [0.6–5.1]	0.3269	2.7 [0.8–9.3]	0.1197
Postop. stoma vs. no stoma	13.5 [2.0–79.6]	**0.0129**	14.0 [1.8–86.3]	**0.0157**

*CI* confidence interval, *IBD* inflammatory bowel disease, *Infl.* inflammatory, *postop.* postoperative

### Stoma vs. no stoma

**Fig. 4:**
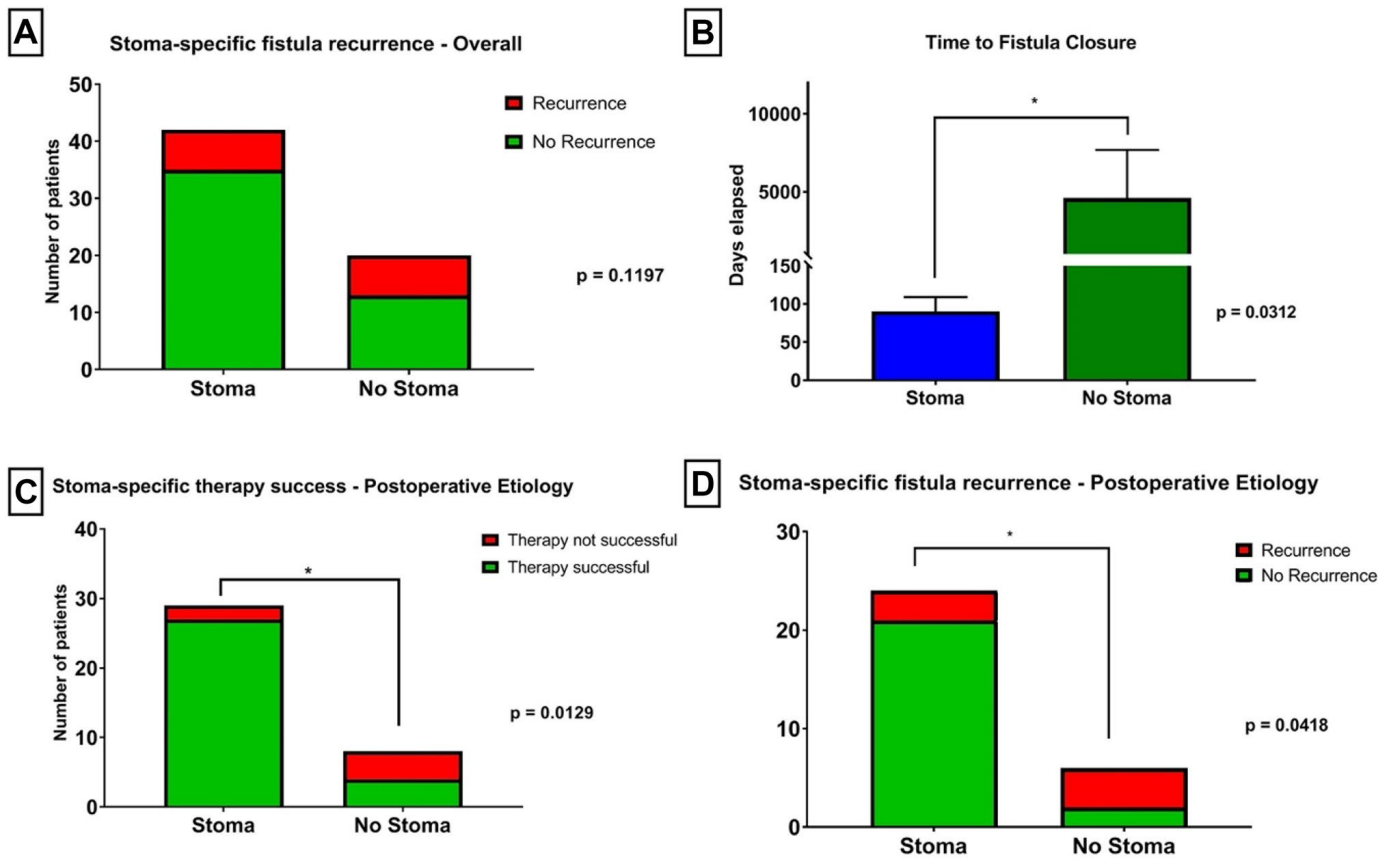
Outcome of fistula-specific therapy, stratified for application of a diverting ostomy. **A** Patients treated with supportive ostomy showed no significant benefit for fistula recurrence (*p* = 0.1197). **B** Application of a supportive ostomy led to significantly lowered time to fistula closure (*p* = 0.0312). In patients with postoperatively derived fistula, application of supportive ostomy showed significantly higher rates of primary fistula closure (**C**; *p* = 0.0129) and lower probability of fistula recurrence (**D**; *p* = 0.0418)

Overall analysis did not reveal an advantage for a temporary stoma with regard to fistula closure rates (Fig. 4A). However, there was a tendency towards lower recurrence rates in case of a temporary stoma. Besides, fistula closure was achieved significantly faster in case of a temporary enteral diversion (*p* = 0.0312; Fig. 4B). Especially the group of postoperative fistulas showed a clear and significant benefit for both, primary fistula closure and recurrence in case of an applied stoma. This was independent of the treatment regimen chosen otherwise (Fig. 4C, D). Details on the effect of a temporary enteral diversion for fistula closure and recurrence rates are shown in Fig. 4, as well as Tables 5 and 6.

**Table 6:** Description of outcome parameter of closure treatment of enterovaginal fistulas, stratified for application of a supportive ostomy

Outcome parameters of fistula therapy	All, *N* (%)/mean [95% CI]	Supportive ostomy		*p* value (stoma vs. no stoma)
		With stoma *N* (%)/mean [95% CI]	Without stoma *N* (%)/mean [95% CI]	
**Therapy success of fistula closure**				0.3269
Successful	62 (67.4%)	43 (64.2%)	19 (76.0%)	
Not successful	30 (32.6%)	24 (35.8%)	6 (24.0%)	
Total	92 (100%)	67 (72.8%)	25 (27.2%)	
**Recurrence of fistula**				0.1197
Recurrence	14 (22.6%)	7 (16.7%)	7 (35.0%)	
No recurrence	48 (77.4%)	35 (83.3%)	13 (65.0%)	
Total	62 (100%)	42 (67.7%)	20 (32.3%)	
**Time to fistula closure (d)**				
	1468 [− 469.1–3405]	90.3 [52.5–128.1]	4585 [1922–11093]	**0.0312**

*CI* confidence interval, *d* days

## Discussion

The presented study analyzes a large cohort of patients with enterovaginal fistulas. This condition, independent of its genesis, leads to crucial impairment of women’s quality of life and often is a challenge with frequent need for several therapeutic interventions. The aim of the presented study was to analyze therapeutic success independent of fistula genesis, to evaluate specific surgical techniques, and to identify potential predictors on surgical outcome.

The presented patient population is heterogenous as enterovaginal fistulas are caused by several underlying conditions. With an overall closure rate of 67.4%, our own results are comparable or better than previously reported in the literature [2, 21–23]. Differences might be due to differences in fistula origin between our cohort and that reported previously [23]. In our analysis, we demonstrate that in case of postoperative fistulas, therapy success is likely and even especially favorable after preceding gynecological surgeries. Postpartum fistulas, in our own patient population had a success closure-rate of 100%, the results being underlined by a previously published series with similar closure rates [24]. Favorable results in non-IBD associated inflammatory by radical treatment of the infectious focus alone, are in line with previous reports [18, 21]. Tumor- and radiation-associated fistulas were found to have the most unfavorable closure rates, just like IBD-associated fistulas with impaired therapeutic success.

Concerning different treatment regimens, more invasive treatment options like an abdominal surgical approach showed the best results even in cases of recurrent fistula. This might also include discontinuity resections with secondary reconstruction at a later point. These results are concordant with data reported by Corte et al. in 2015, who also found higher rates of fistula closure when major surgical procedures were preferred [21]. Similarly, a local surgical approach was likely to lead to therapy success as well. Even though our results suggest a rather radical treatment to show higher association with satisfying fistula closure and recurrence rates, the time point of using invasive treatments within the curative therapy sequence does not seem to be important. Therefore, the primary use of less invasive treatment options might still be applicable for selected patients, especially for those with higher comorbidities. Although an older study by Lowry and co-workers found two or more prior surgical approaches for fistula closure to be associated with a higher relative risk for recurrence, our own data still suggests that in case of initial treatment failure, the use of a more invasive therapy is still helpful and reasonable at a later point without significant impairment in outcome [22]. Thus, we advocate an individualized step-up approach, rather than a maximal invasive treatment in all patients for treatment of enterovaginal fistulas.

Whereas Corte and colleagues identified a diverting stoma as an independent factor for treatment success in case of rectovaginal fistula, we did not confirm this [21]. Nevertheless, our results suggest that a temporary stoma shortens time to fistula closure. However, this was barely significant with regard to the whole population and could not be concluded in a subgroup-analysis. The latter seems mainly caused by large differences in the time intervals until fistula closure. Patients with a postoperative fistula profited most from a stoma whereas patients with inflammatory genesis, such as diverticulitis, did not in the same manner.

Among patients with unsuccessful treatment attempts, the CCI score was higher. This finding is in line with previous results as a higher CCI is a risk factor for anastomotic leakage in colorectal surgery [25, 26]. Therefore, in case of a high CCI score in patients with enterovaginal fistula, the treatment regime has to be planned carefully, and emphasis has to be put on optimization of comorbidities prior to treatment.

There are some limitations of this study. The retrospective character does not allow for standardization of the documentation of fistula characteristics, such as height and length. However, a prospective study with regard to therapy strategies for enterovaginal fistulas might be impossible to realize since many individual aspects play a substantial role in the therapeutic decision-making. Especially heterogeneity of the disease and patient cohort leads to a variety of treatment options. The presented patient population differs to some extent from those previously published, e.g., with a much lower proportion of postpartum fistulas. However, the analysis of this large patient population—as compared to previous studies—allowed to perform subgroup analysis concerning etiology-specific aspects and success rate of different treatment approaches.

Our study substantiates the multifarious character of enterovaginal fistulas regarding their genesis, as well as possible therapeutic options, and strengthens the fact that the therapeutic approach needs to be tailored individually according to fistula etiology, localization, and individual patient characteristics [2, 14, 23, 27, 28].

**Fig. 5:**
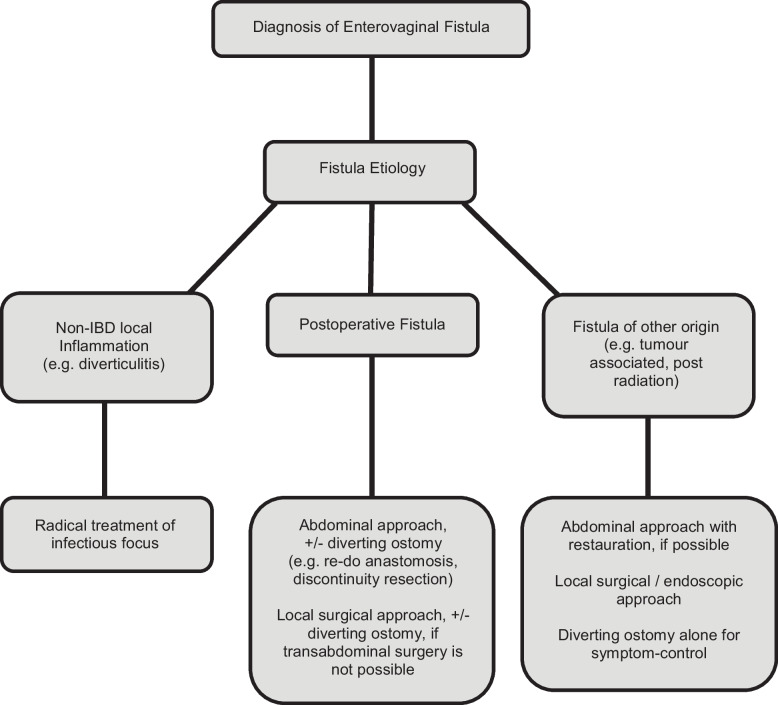
Flow chart on possible treatment approaches for enterovaginal fistula

The results suggest to favor invasive treatment options, as well as the use of a diverting stoma, in order to achieve beneficial therapy success and low recurrence rates. In detail, the results of our study propose that in case of inflammatory origin of the fistula, surgical treatment of the infectious focus alone will likely lead to success. In case of postoperative enterovaginal fistulas a radical abdominal re-operation including a temporary stoma is most favorable with high closure-rates and will shorten the time interval to fistula closure. If this cannot be performed, a local surgical intervention will also likely lead to therapeutic success and should be favored over the sole use of endoscopic or conservative treatment options. For those patients, an individual step-up approach (local treatment first, a more radical approach in case of fistula recurrence) is a valuable alternative treatment option (Fig. 5).

## Data Availability

Not applicable
